# Minimal Residual Disease Detected by the 7NB-mRNAs ddPCR Assay Is Associated with Disease Progression in High-Risk Neuroblastoma Patients: A Prospective Multicenter Observational Study in Japan

**DOI:** 10.3390/biology12101350

**Published:** 2023-10-20

**Authors:** Noriyuki Nishimura, Toshiaki Ishida, Isao Yokota, Kimikazu Matsumoto, Hiroyuki Shichino, Hiroyuki Fujisaki, Takeo Sarashina, Takehiko Kamijo, Tetsuya Takimoto, Tomoko Iehara, Tatsuro Tajiri, on behalf of the JCCG Neuroblastoma Committee

**Affiliations:** 1 Department of Public Health, Graduate School of Health Science, Kobe University, Kobe 654-0142, Japan; 2 Department of Hematology/Oncology, Kobe Children’s Hospital, Kobe 650-0047, Japan; ishida_kch@hp.pref.hyogo.jp; 3 Department of Biostatistics, Faculty of Medicine, Hokkaido University, Sapporo 060-0808, Japan; yokotai@pop.med.hokudai.ac.jp; 4 Children’s Cancer Center, National Center for Child Health and Development, Tokyo 157-8535, Japan; matsumoto-kmk@ncchd.go.jp; 5 Department of Pediatrics, National Center for Global Health and Medicine, Tokyo 162-8655, Japan; hshichino@hosp.ncgm.go.jp; 6 Department of Pediatric Hematology/Oncology, Osaka City General Hospital, Osaka 534-0021, Japan; h-fujisaki@med.osakacity-hp.or.jp; 7 Department of Pediatrics, Asahikawa Medical University, Asahikawa, 078-8510, Japan; sara5p@asahikawa-med.ac.jp; 8 Research Institute for Clinical Oncology, Saitama Cancer Center, Saitama 362-0806, Japan; tkamijo@saitama-pho.jp; 9 Department of Childhood Cancer Data Management, National Center for Child Health and Development, Tokyo 157-8535, Japan; takimoto-t@ncchd.go.jp; 10 Department of Pediatrics, Graduate School of Medical Science, Kyoto Prefectural University of Medicine, Kyoto 602-8566, Japan; iehara@koto.kpu-m.ac.jp; 11 Department of Pediatric Surgery, Faculty of Medical Sciences, Kyushu University of Medicine, Fukuoka 812-8582, Japan; taji@pedsurg.med.kyushu-u.ac.jp

**Keywords:** neuroblastoma (NB), minimal residual disease (MRD), bone marrow (BM), peripheral blood (PB), neuroblastoma-associated mRNAs (NB-mRNAs), droplet digital PCR (ddPCR)

## Abstract

**Simple Summary:**

Neuroblastoma (NB) is a common pediatric tumor, and less than 50% of children with high-risk (HR)-NB can achieve long-term survival. This is mainly due to a tumor relapse caused by the activation of therapy-resistant minimal residual disease (MRD). Several assays measuring different sets of MRD markers by quantitative PCR (qPCR) or droplet digital PCR (ddPCR) were reported to have a significant prognostic value for MRD in HR-NB patients. The 7NB-mRNAs ddPCR assay was reported to outperform other qPCR assays by a retrospective in-house observational study. In the present study, the Japan Children’s Cancer Group (JCCG) Neuroblastoma Committee conducted a prospective multicenter observational study to evaluate a prognostic value of MRD in bone marrow (BM-MRD) and peripheral blood (PB-MRD) measured by the 7NB-mRNAs ddPCR assay. A total of 19 BM and 19 PB samples were collected from seven HR-NB patients. BM-MRD and PB-MRD estimated area under curve (AUC) of 0.767 and 0.800 with a significant accuracy (AUC > 0.7), validating a prognostic value of BM-MRD obtained by a previous study (AUC 0.723). The present study will pave the way to introduce a MRD assay into HR-NB patients’ clinical practice.

**Abstract:**

High-risk neuroblastoma (HR-NB) patients remain far from obtaining optimal outcomes, with more than 50% relapse/regrowth rate despite current intensive multimodal therapy. This originated from the activation/proliferation of chemoresistant minimal residual disease (MRD). MRD with a significant prognostic was reported by several quantitative PCR (qPCR) or droplet digital PCR (ddPCR) assays quantitating different sets of NB-associated mRNAs (NB-mRNAs). The 7NB-mRNAs ddPCR assay quantitating CRMP1, DBH, DDC, GAP43, ISL1, PHOX2B, and TH mRNAs was reported to outperform other qPCR assays by a retrospective in-house observational study. In the present study, the Japan Children’s Cancer Group (JCCG) Neuroblastoma Committee conducted a prospective multicenter observational study aimed at evaluating a prognostic value of MRD in bone marrow (BM-MRD) and peripheral blood (PB-MRD) detected by 7NB-mRNAs ddPCR assay. Between August 2018 and August 2022, 7 HR-NB patients who registered for JCCG clinical trials (JN-H-11 and JN-H-15) were enrolled. A total of 19 BM and 19 PB samples were collected, and 4/15 BM and 4/15 PB samples were classified as progressive disease (PD)/non-PD samples. BM-MRD and PB-MRD estimated area under curve (AUC) of 0.767 and 0.800 with a significant accuracy (AUC > 0.7). The present study validated a prognostic value of BM-MRD obtained by a previous study (AUC 0.723) and revealed the significant accuracy of PB-MRD as well as BM-MRD.

## 1. Introduction

Neuroblastoma (NB) is almost exclusively a cancer of children, which accounts for approximately 10% of all pediatric cancer and 15% of all pediatric cancer deaths [1,2]. It originates from neural crest-derived cells and shows a remarkable heterogeneity ranging from spontaneous regression to malignant progression [3,4]. Newly diagnosed NB patients have very different prognoses and are risk-stratified into low-risk (LR), intermediate-risk (IR), and high-risk (HR) groups [5,6,7]. Although LR- and IR-NB patients have excellent outcomes with near uniform survival, less than 50% of HR-NB patients will achieve long-term survival [8]. HR-NB patients are treated by an intensified multimodal therapy composed of induction chemotherapy (IC), high-dose chemotherapy (HDC) with autologous peripheral blood stem cell (PBSC) transplantation, surgery, radiotherapy, and immunotherapy, and nearly 80% of them achieve remission during induction chemotherapy [9]. However, most HR-NB patients have a minimal residual disease (MRD) that may cause relapse, and more than half of HR-NB patients actually experience relapse with a dismal outcome [8]. An accurate evaluation of MRD in HR-NB patients is essential to monitor the treatment response and disease burden for an optimal outcome.

Because NB is deficient in recurrent genomic aberrations, the current MRD evaluation in HR-NB patients uses a set of neuroblastoma-associated mRNAs (NB-mRNAs) to detect NB cells in bone marrow (BM) and peripheral blood (PB) samples [10,11]. Among a growing number of assays evaluating MRD in BM (BM-MRD) and PB (PB-MRD), several assays quantitating different but overlapping sets of NB-mRNAs by quantitative PCR (qPCR) or droplet digital PCR (ddPCR) are reported to have a significant prognostic value of BM-MRD and PB-MRD for HR-NB patients [12,13,14,15,16,17].

The 7NB-mRNAs ddPCR assay quantitating CRMP1, DBH, DDC, GAP43, ISL1, PHOX2B, and TH mRNAs (AUC = 0.723) was shown to be more accurate than the 5NB-mRNAs qPCR assay quantitating CHGA, DCX, DDC, PHOX2B, and TH mRNAs (AUC = 0.622) by a retrospective in-house observational study [16,17]. Although BM-MRD measured by the 7NB-mRNAs ddPCR assay was significantly associated with relapse in an observational study cohort, the further validation in an independent cohort was required. To validate a prognostic value of the 7NB-mRNAs ddPCR assay, the Japan Children’s Cancer Group (JCCG) Neuroblastoma Committee conducted a prospective multicenter observational study of BM-MRD and PB-MRD in HR-NB patients enrolled in the JCCG JN-H-11 (UMIN000005045) or JN-H-15 (UMIN000016848) clinical trial. In the present study, we performed the 7NB-mRNAs ddPCR assay on a total of 19 BM and 19 PB samples collected from seven enrolled HR-NB patients and evaluated its clinical impact.

## 2. Materials and Methods

### 2.1. Study Design and Patient Samples

This study was a prospective multicenter observational study assessing the prognostic value of BM-MRD and PB-MRD detected by the 7NB-mRNAs ddPCR assay in HR-NB patients. Eligible patients were enrolled in the JCCG JN-H-11 (UMIN000005045) or JN-H-15 (UMIN000016848) clinical trial and completed or discontinued the protocol therapy. This study was open for patient registration from August 2018 to August 2021, and for observation from August 2021 to August 2022. Patient samples were collected as residual samples of standard clinical practice. The primary endpoint was positive predictive value (PPV) and negative predicted value (NPV) of BM-MRD and PB-MRD. In this study, PPV and NPV were defined as follows: PPV = the number of PD samples/the number of high MRD (≥cut-off value) samples, NPV = the number of non-PD samples/the number of low MRD (<cut-off value) samples. This study was approved by the Institutional Review Boards of the Kobe University Hospital (No. 180133) and the participating institutions, and was conducted in accordance with the ethical principles of the Declaration of Helsinki. Written informed consent was obtained from all participants or their guardians upon enrollment.

### 2.2. Patient Sample Processing and MRD Analysis

Patient sample processing and MRD analysis were conducted as described previously [17]. Briefly, nucleated cells were collected from BM and PB samples using Lymphoprep (Abbott Diagnostics Technologies, Oslo, Norway). Collected cells were subjected to total RNA extraction with a TRIzol Plus RNA purification kit (Life Technologies, Carlsbad, CA, USA). Total RNA (1 or 0.5 μg) was then subjected to cDNA synthesis with a Quantitect reverse transcription kit (Qiagen, Valencia, CA, USA). Expression of 7 NB-mRNAs (CRMP1, DBH, DDC, GAP43, ISL1, PHOX2B, and TH) and a reference gene mRNA (HPRT1) was determined with a Universal Probe Library (Roche, Mannheim, Germany) using a QX200 ddPCR system (Bio-Rad Laboratories, Hercules, CA, USA) according to the digital Minimum Information for Publication of Quantitative Digital PCR Experiments (MIQE) guideline (Table S1) [18,19]. For the MRD evaluation, the weighted sum of 7 relative copy numbers (level of each NB-mRNA), in which the reciprocal of 90 percentile in non-NB control samples, was calculated and designated as 7NB-mRNAs (combined signature).

### 2.3. Disease Evaluation

Disease evaluation was performed in accordance with the International Neuroblastoma Response Criteria (INRC) [20,21], based on the available medical records as described previously [17]. Disease status assigned was either “progressive disease (PD)” that corresponded to progressive disease or “non-progressive disease (non-PD)” that corresponded to complete response, very good partial response, partial response, mixed response, or no response. In this study, disease evaluation was conducted at 0 months, 6 months, and over 12 months after every BM and PB sample collection, and all BM and PB samples were classified as PD or non-PD sample.

### 2.4. Statistical Analysis

For each patient, the levels of BM-MRD/PB-MRD at all evaluated times were plotted. The sensitivity, specificity, PPV, and NPV between the levels of BM-MRD/PB-MRD and PD were calculated. The receiver operator characteristic (ROC) curves are plotted for the levels of BM-MRD/PB-MRD against PD, and their area under the curve (AUC) were calculated. For statistical analyses, a modified version of R commander EZR (version 1.55, www.jichi.ac.jp/saitama-sct/SaitamaHP.files/statmedEN.html, accessed on 1 September 2022; Saitama Medical Center, Jichi Medical University, Saitama, Japan) was used [22].

## 3. Results

### 3.1. Characteristics of Patients and Samples

To assess a prognostic value of BM-MRD and PB-MRD detected by the 7NB-mRNAs ddPCR assay, the JCCG Neuroblastoma Committee conducted a prospective multicenter observational study. A total of seven HR-NB patients were enrolled in this study at a median of 31 (range 16–87) months after diagnosis and observed for a median of 33 (range 14–47) months. As summarized in Table 1, the enrolled patients showed the typical characteristics of HR-NB: a median of 42 months old at diagnosis (range 30–75 months), 86% (6/7) with an adrenal gland tumor, 29% (2/7) with a MYCN-amplified tumor, 71% (5/7) with BM metastasis at diagnosis, and 86% (6/7) with INSS stage 4. A total of 19 BM and 19 PB samples were collected from the seven enrolled patients, with a median of 2 (range 1–7) samples per patient.

**Figure 1:**
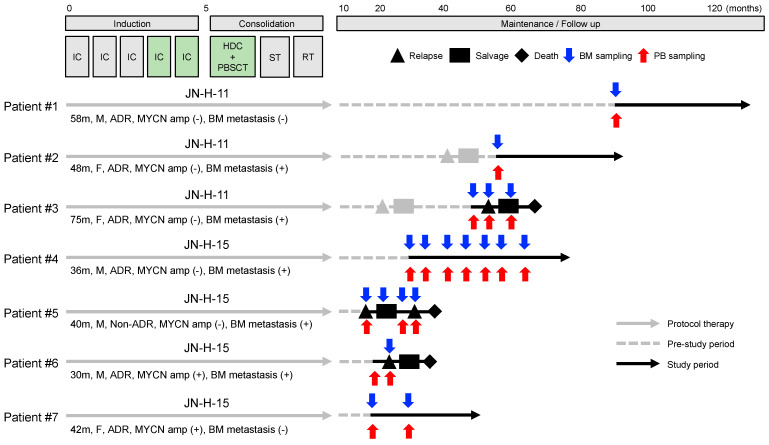
Clinical course of enrolled HR−NB patients. All patients completed JN−H−11 or JN−H−15 protocol therapy before registration. The fourth and fifth cycles of IC and HDC regimens were 05A3, 05A3, and MEC for JN−H−11 protocol and ICE, ICE, and BU+LPAM for JN−H−15 protocol, respectively. In total, 2 patients relapsed in the pre-study period and 3 patients in the study period. A total of 4 patients were alive and 3 patients dead. IC, induction chemotherapy; HDC, high-dose chemotherapy; PBSCT, peripheral blood stem cell transplantation; ST, surgical therapy, RT, radiation therapy; BM, bone marrow, PB, peripheral blood; m, months; M, male; F, female; ADR, adrenal gland tumor; MYCN, MYCN proto-oncogene bHLH transcription factor.

In this study, three and four patients were treated with JN-H-11 and JN-H-15 protocols, respectively. JN-H-11 and JN-H-15 protocols adopted the same treatment backbone as JN-H-07 except for the timing of surgery [23]. The treatment backbone consisted of five cycles of IC and HDC with autologous PBSC transplantation followed by surgical resection of the primary tumor (delayed local surgery) and radiotherapy administered to the primary tumor site. The difference between JN-H-11 (UMIN000005045) and JN-H15 (UMIN000016848) protocols was the fourth and fifth cycles of IC and HDC regimens: JN-H-11 (first IC 05A1 (CPA + VCR + THP + CDDP), second IC 05A3 (CPA + VCR + THP + CDDP), third IC 05A3, 4th IC 05A3, fifth IC 05A3, and HDC MEC (LPAM + VP-16 + CBDCA) regimens) and JN-H-15 (first IC 05A1, second IC 05A3, third IC 05A3, fourth IC ICE (IFO + CBDCA + VP-16), fifth IC ICE and HDC BU + LPAM regimens). As illustrated in Figure 1, all seven patients completed JN-H-11 or JN-H-15 protocol therapy before registration. A total of six relapses occurred in four patients: two relapses before registration, one relapse at registration, and three relapses during observation. At the end of this study, four patients were alive and three patients dead.

### 3.2. Expression Levels of Each NB-mRNA and 7NB-mRNAs in the BM and PB Samples

**Figure 2:**
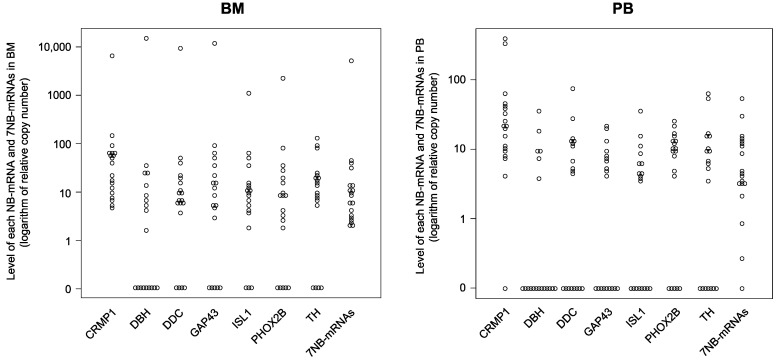
Levels of each NB-mRNA and the 7NB-mRNAs in BM and PB samples. The levels of each NB-mRNA and 7NB-mRNAs in 19 BM and 19 PB samples were determined by ddPCR. BM, bone marrow; PB, peripheral blood; NB-mRNA, neuroblastoma-associated mRNA; CRMP1, collapsin response mediator protein 1; DBH, dopamine beta-hydroxylase; DDC, dopa decarboxylase; GAP43, growth-associated protein 43; ISL1, ISL LIM homeobox 1; PHOX2B, paired-like homeobox 2b; TH, tyrosine hydroxylase.

In this study, a total of 19 BM and 19 PB samples were subjected to 7NB-mRNAs ddPCR assay [17]. Consistent with a retrospective in-house observational study [17], the levels of each NB-mRNA (CRMP1, DBH, DDC, GAP43, ISL1, PHOX2B, and TH) and 7NB-mRNAs in BM (median: 1.64–37.65) and PB (median: 0–20.77) samples substantially varied between patients and sample collection timing, respectively (Table 2). As shown in Figure 2, the 7NB-mRNAs were more sensitively detected than each NB-mRNA in the BM (7NB-mRNAs: 100%, each NB-mRNA: 53–100%) and the PB (7NB-mRNAs: 95%, each NB-mRNA: 19–95%) samples.

### 3.3. BM-MRD and PB-MRD Detected by 7NB-mRNAs ddPCR Assay

**Figure 3:**
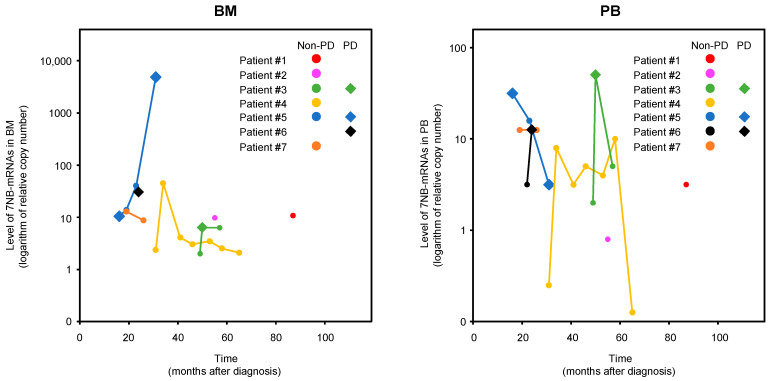
BM-MRD and PB-MRD detected by 7NB-mRNAs ddPCR assay. BM-MRD and PB-MRD of each enrolled patient (#1–#7) were dot-plotted with time (months after diagnosis) and disease evaluation at sample collection (observation 0 months). BM, bone marrow; PB, peripheral blood; NB-mRNA, neuroblastoma-associated mRNA; PD, progressive disease.

BM-MRD and PB-MRD of each enrolled patient (#1–#7) were dot-plotted with time (months after diagnosis) and disease evaluation (PD or non-PD) at sample collection (observation 0 months) in Figure 3. Among a total of 19 BM-MRD 19 PB-MRD, their highest and lowest values were evaluated as PD and non-PD, respectively, as expected. Although BM-MRD and PB-MRD of each enrolled patient showed a very dynamic change over time, they tended to become elevated in PD samples compared to non-PD samples. When PD samples were examined as pairs of BM-MRD and PB-MRD, all four PD sample pairs of patients #3, #5, and #6 showed the markedly high values: PB-MRD at 54 months was markedly high in patient #3, PB-MRD at 18 months and BM-MRD at 34 months in patient #5, and PB-MRD at 26 months in patient #6 (Figure 3).

### 3.4. ROC Analysis of BM-MRD and PB-MRD for Disease Progression

**Figure 4:**
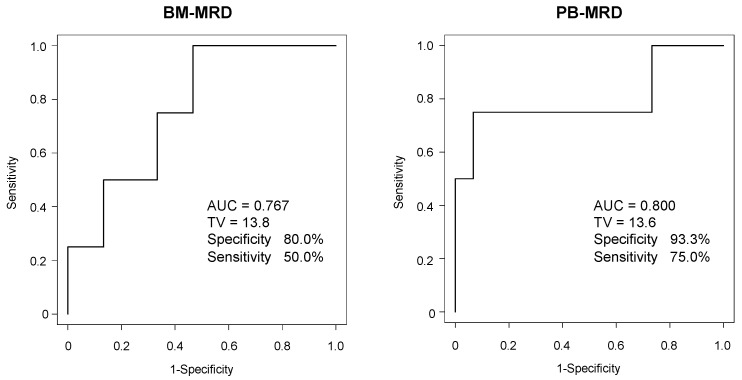
ROC analysis of BM-MRD and PB-MRD for disease progression. The ROC curves were plotted for BM-MRD and PB-MRD at sample collection (observation 0 months) for disease progression (4 PD and 15 non-PD). AUC, the area under curve; TV, threshold value.

Because BM-MRD and PB-MRD substantially differed between PD and non-PD, the ROC curves were plotted for BM-MRD and PB-MRD at sample collection (observation 0 months) and for disease progression (four PD and fifteen non-PD) in Figure 4. It estimated an AUC of 0.767 with significant accuracy (>0.7) for BM-MRD and an AUC of 0.800 with significant accuracy (>0.7) for PB-MRD, respectively, demonstrating that BM-MRD and PB-MRD were significantly associated with disease progression in this study samples.

### 3.5. PPV and NPV of BM-MRD and PB-MRD

To access the PPV and NPV, the cut-off values were set as 15 for BM-MRD and 13 for PB-MRD according to the ROC curves (Figure 4). All 19 BM and PB samples were then stratified by MRD and disease evaluations. MRD evaluation resulted in four BM-MRD-positive (≥15)/15 BM-MRD-negative (<15) samples and four PB-MRD-positive (≥13)/15 PB-MRD-negative (<13) samples, respectively. Disease evaluation at 0, 6, and over 12 months resulted in 4 PD/15 non-PD, 7 PD/12 non-PD, and 8 PD/11 non-PD for BM samples and 4 PD/15 non-PD, 8 PD/11 non-PD, and 8 PD/11 non-PD for PB samples, respectively. Accordingly, PPV and NPV were calculated as 50–75% and 67–87% for BM-MRD and 75–100% and 73–93% for PB-MRD (Table 3).

## 4. Discussion

In the present prospective multicenter observational study, we evaluated BM-MRD and PB-MRD detected by the 7NB-mRNAs ddPCR assay in a total of 19 BM and 19 PB samples collected from seven enrolled patients and validated a prognostic value of BM-MRD reported in a previous retrospective in-house observational study [17]. In addition, this study revealed a compatible prognostic value of PB-MRD (AUC 0.800) with BM-MRD (AUC 0.767).

The two independent cohorts (73 samples from 15 patients, with a median of four samples per patient and nineteen samples from seven patients, with a median of two samples per patient) consistently provided a significant accuracy (AUC 0.723 and AUC 0.767) of BM-MRD detected by the 7NB-mRNAs ddPCR assay [17]. Both cohorts collected BM samples after the completion of high-dose chemotherapy with autologous PBSC transplantation, surgery, and radiotherapy, whereas BM samples at/after relapse (PD) were included only in the present cohort. This is a unique characteristic of the present JCCG study collecting both BM and PB samples prospectively in multiple institutions and brings us two novel findings. First, a prospective disease evaluation for every BM and PB sample enabled us to analyze the timing of MRD-predicted disease progression and revealed that MRD-predicted disease progression occurred within 12 and 6 months after BM and PB sample collection, respectively (Table 3). Although the number of PD samples is very limited, it is tempting to speculate that PB-MRD predicts disease progression earlier than BM-MRD. Second, simultaneous monitoring of BM-MRD and PB-MRD enabled us to become aware of their interrelation. Although it is naturally expected that BM-MRD and PB-MRD become elevated when disease progression occurs in each patient, they are not always higher in PD samples than in non-PD samples (Figure 3). Paired examination of BM and PB samples revealed that PD sample pairs showed markedly high BM-MRD or PB-MRD. Although further validation is required, it may reflect that MRD dynamically changes its preferential location between BM and PB.

Although NB cells were detected in PB three decades ago [24,25], only a few reports showed a significant prognostic value of PB-MRD [13,15,16]. This was mainly due to the prevailing recognition in the clinics that PB-MRD was 10–100 times lower than BM-MRD [16,17] and MRD was difficult to detect in PB samples. However, it was not always true and depended on the timing of BM and PB sample collection. PB-MRD was almost 100 times lower than BM-MRD at initial diagnosis but occasionally became higher than BM-MRD during induction and consolidation therapy [17]. In the present study, PB-MRD was higher than BM-MRD in seven out of nineteen BM and PB sample pairs, and demonstrated a significant prognostic value (AUC 0.800) that was compatible with BM-MRD (AUC 0.767, Figure 4). It warrants further study to validate a prognostic value of PB-MRD detected by the 7NB-mRNAs ddPCR assay in an independent cohort with a larger number of patients and samples.

However, the small number of patients (*n* = 7) and samples (*n* = 19) was an apparent limitation of the present study. To analyze the prognostic impact of MRD for HR-NB patients, we used the previous JCCG JN-H-11 (UMIN000005045) and JN-H-15 (UMIN000016848) clinical trials. This was because we had tried to recruit as many patients as possible, who were expected to experience PD events within a 1–2 year period. The eligibility was set as the enrollment in these clinical trials and the completion/discontinuation of these protocol therapies. As a consequence, this research design made the participation in the present study difficult. We are now conducting the current JCCG JN-H-20 (jRCTs041210034) clinical trial creating another independent cohort to analyze a clinical value of MRD.

## 5. Conclusions

In summary, we have validated a prognostic value of BM-MRD detected by ddPCR with 7NB-mRNAs in a prospective multicenter observational study independent of a previous in-house observational study [17] and revealed a significant accuracy of PB-MRD as well as BM-MRD.

## Figures and Tables

**Table 1:** Characteristics of patients and samples.

Enrolled Patients	
Treatment protocol	
JN-H-11	3
JN-H-15	4
Registration (months after initial diagnosis)	
Median	31
Range	16–87
Observation period (months)	
Median	33
Range	14–47
Disease evaluation at registration	
PD	1
Non-PD	6
Outcome	
Alive	4
Dead	3
Age at diagnosis (months)	
Median	42
Range	30–75
Sex	
Male	4
Female	3
Primary tumor site	
Adrenal gland	6
Non-adrenal gland	1
MYCN status	
Amplified	2
Non-amplified	5
BM metastasis at diagnosis	
Positive	5
Negative	2
INSS stage at diagnosis	
Stage 3	1
Stage 4	6
Collected samples	
BM samples	Total: 19 Per patient: 1–7 (Median 2)
PB samples	Total: 19 Per patient: 1–7 (Median 2)

PD, progressive disease; MYCN, MYCN proto-oncogene bHLH transcription factor; INSS, International Neuroblastoma Staging System; BM, bone marrow; PB, peripheral blood.

**Table 2:** Expression levels of each NB-mRNA and 7NB-mRNAs in the BM and PB samples.

	BM Samples				PB Samples			
	Range	Median	IQR	*n*	Range	Median	IQR	*n*
CRMP1 mRNA	4.71–6867.47	37.65	10.55–62.86	19	0.00–396.83	20.77	9.93–40.53	19
DBH mRNA	0.00–14,337.35	1.64	0.00–11.08	19	0.00–35.44	0.00	0.00–5.55	19
DDC mRNA	0.00–9530.12	6.73	4.69–17.08	19	0.00–75.95	4.73	0.00–12.53	19
GAP43 mRNA	0.00–12,289.16	8.82	1.43–27.32	19	0.00–21.24	4.14	0.00–7.49	19
ISL1 mRNA	0.00–1168.67	9.94	3.94–14.31	19	0.00–36.73	3.88	0.00–6.15	19
PHOX2B mRNA	0.00–2216.87	8.07	0.94–17.53	19	0.00–25.32	9.52	2.07–13.36	19
TH mRNA	0.00–124.26	13.04	6.08–21.18	19	0.00–63.10	6.18	0.00–12.63	19
7NB-mRNAs	2.00–4948.76	8.67	3.23–13.37	19	0.00–51.95	4.60	3.13–12.71	19

NB-mRNA, neuroblastoma-associated mRNA; BM, bone marrow; PB, peripheral blood; IQR, interquartile range; CRMP1, collapsin response mediator protein 1; DBH, dopamine beta-hydroxylase; DDC, dopa decarboxylase; GAP43, growth-associated protein 43; ISL1, ISL LIM homeobox 1; PHOX2B, paired-like homeobox 2b; TH, tyrosine hydroxylase.

**Table 3:** PPV and NPV of BM-MRD and PB-MRD.

Observation	BM-MRD Cut off = 15		PB-MRD Cut off = 13	
	PPV	NPV	PPV	NPV
0 M	50% (2/4)	87% (13/15)	75% (3/4)	93% (14/15)
6 M	75% (3/4)	73% (11/15)	100% (4/4)	73% (11/15)
>12 M	75% (3/4)	67% (10/15)	100% (4/4)	73% (11/15)

PPV, positive predictive value; NPV, negative predictive value; M, months.

## Data Availability

The data generated and analyzed in this study are available upon reasonable request.

## Supplementary Materials

The following supporting information can be downloaded at: https://www.mdpi.com/article/10.3390/biology12101350/s1, Table S1: Primer sequences and Universal Probe Library numbers.
