# Supplementary material for: Minimal Residual Disease Detected by the 7NB-mRNAs ddPCR Assay Is Associated with Disease Progression in High-Risk Neuroblastoma Patients: A Prospective Multicenter Observational Study in Japan
Source: Biology (Basel). 2023 Oct 20;12(10):1350. doi: 10.3390/biology12101350 (PMC10604505; doi:10.3390/biology12101350)
Supplement: Supplementary file 1 [file biology-12-01350-s001.zip › biology-2557959-supplementary.pdf]

Table S1. Primer sequences and Universal Probe Library numbers

Gene name	Primer sequences	Universal Probe Library numbers
CRMP1<br>NM_001014809	5'-CCAATCCCTTTATGCTGACG-3' (sense)<br>5'-GGAACGATTAAGTTCTCTCCTATTTG-3' (antisense)	No. 65
DBH<br>NM_000787	5'-TGGGGACACTGCCTATTTTG-3' (sense)<br>5'-TTCTGGGGTCCTCTGCAC-3' (antisense)	No. 3
DDC<br>NM_000790	5'-CTGGAGAAGGGGGAGGAGT-3' (sense)<br>5'-GCCGATGGATCACTTTGGT-3' (antisense)	No. 49
GAP43<br>NM_002045	5'-GAGGATGCTGCTGCCAAG-3' (sense)<br>5'-GGCACTTTCCTTAGGTTTGGT-3' (antisense)	No. 26
ISL1<br>NM_002202	5'-AAGGACAAGAAGCGAAGCAT-3' (sense)<br>5'-TTCCTGTCATCCCCTGGATA-3' (antisense)	No. 66
PHOX2B<br>NM_003924	5'-CTACCCCGACATCTACACTCG-3' (sense)<br>5'-CTCCTGCTTGCGAAACTTG-3' (antisense)	No. 17
TH<br>NM_199292	5'-TCAGTGACGCCAAGGACA-3' (sense)<br>5'-GTACGGGTCGAACTTCACG-3' (antisense)	No. 42
HPRT1<br>NM_000194	5'-TGACCTTGATTTATTTTGCATACC-3' (sense)<br>5'-CGAGCAAGACGTTCAGTCCT-3' (antisense)	No. 73
